# Successful Resolution of a Large Left Atrial and Left Atrial Appendage Thrombus with Rivaroxaban

**DOI:** 10.1155/2019/6076923

**Published:** 2019-04-09

**Authors:** Safwan Gaznabi, Ashraf Abugroun, Hasan Mahbub, Enrique Campos

**Affiliations:** ^1^Department of Internal Medicine, Advocate Illinois Masonic Medical Center, 836 W Wellington Ave., Chicago, IL 60657, USA; ^2^Department of Internal Medicine, Flushing Hospital Medical Center, 4500 Parsons Blvd, Flushing, NY 11355, USA; ^3^Department of Cardiology, Advocate Illinois Masonic Medical Center, 836 W Wellington Ave, Chicago, IL 60657, USA

## Abstract

A 79-year-old male was admitted to the hospital for acute exacerbation of heart failure. The patient had history of atrial fibrillation and was planned for cardioversion. Preprocedure transesophageal echocardiogram (TEE) revealed a large multilobulated mobile thrombus in the left atrial appendage. The patient refused warfarin therapy and instead chose to take rivaroxaban. Upon outpatient follow-up, 3 months later, no visible thrombus was appreciated on repeat TEE. This case demonstrates successful resolution of left atrial and left atrial appendage thrombi with the use of rivaroxaban. At present time, limited data is available to support the use of rivaroxaban for treatment of intracardiac thrombi. This case highlights the need for further studies to investigate the outcomes and relative efficiency of use of direct oral anticoagulants (DOACs) in lysis of intracardiac thrombus. The benefits of DOACs compared to the standard of therapy could increase patient compliance, reduce length of stay, and improve treatment efficacy.

## 1. Introduction

Atrial fibrillation is an ever-growing global problem, with an estimated 2.7-6.1 million affected in the United States and roughly 33.5 million affected worldwide [1]. Left atrial abnormalities, such as dilated left atrium and reduced left atrial and/or left atrial appendage blood flow, are independent risk factors for development of thromboembolism [2, 3]. In order to estimate the annual minimal risk of a thromboembolic event in these patients, we use the CHA2DS2-VASc scoring tool. Anticoagulation therapy helps to mitigate the risks of thromboembolic events and specifically in cases of nonvalvular atrial fibrillation (NVAF) [4, 5]. In cases of left atrial or left atrial appendage (LA/LAA) thrombus, current guidelines recommend vitamin K antagonist (VKA) therapy [6–10]. Here, we describe a successful resolution of left atrial and left atrial appendage thrombus with use of rivaroxaban.

## 2. Case Report

**Figure 1:**
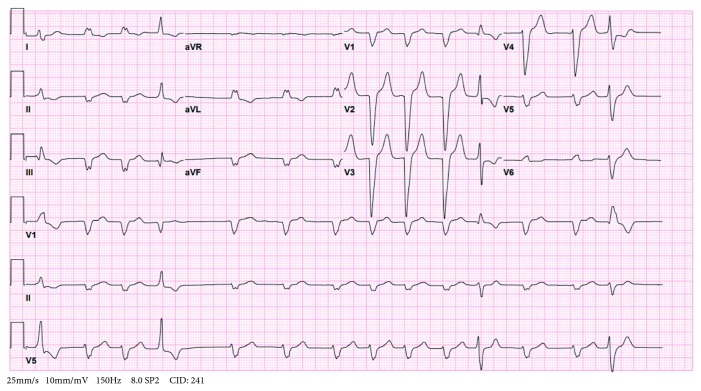
EKG shows atrial fibrillation and LBBB.

**Figure 2:**
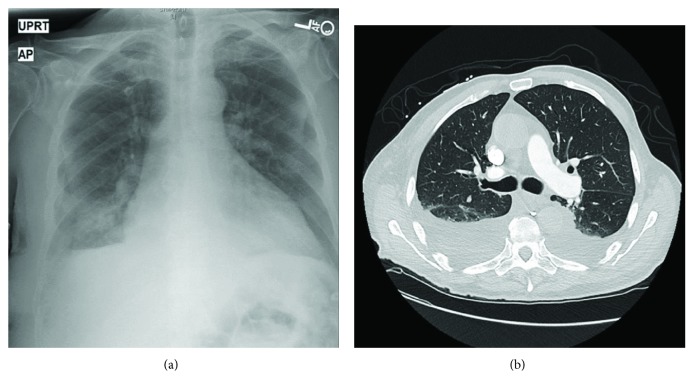
(a) CXR showed cardiomegaly with pulmonary congestion and bilateral pleural effusion. (b) CT chest with contrast showed moderately large bilateral pleural effusions, right greater than left, with cardiomegaly and reflux of IV contrast into the IVC, consistent with cardiogenic pulmonary vascular congestion.

A 79-year-old male with past medical history of hypertension, atrial fibrillation (CHA2DS2-VASc score = 4, only on Aspirin), type 2 diabetes mellitus, and right lower extremity leiomyosarcoma with lymphedema of the affected limb treated with surgical resection and radiotherapy presented to the emergency department with exertional dyspnea, worsening of lower extremity edema, and weight gain. On arrival vitals shows blood pressure 140/95, heart rate 80, and SpO_2_ 98. Physical examination was remarkable for irregular heartbeat, decreased bilateral lung sounds, and bilateral grade 3+ lower extremity edema up to the sacrum. Electrocardiogram (EKG) showed atrial fibrillation with new left bundle branch block (LBBB) (Figure 1). The laboratory workup was significant for brain natriuretic peptide (BNP) 2,233 pg/ml, troponin 0.38 ng/ml, and d-dimer 1.81 mg/l. Otherwise, he had normal basic metabolic panel (BMP) and complete blood count (CBC). Chest X-ray (CXR) and computed tomography (CT) of the chest showed cardiomegaly and moderate pleural effusion in bilateral lung fields (Figure 2).

**Figure 3:**
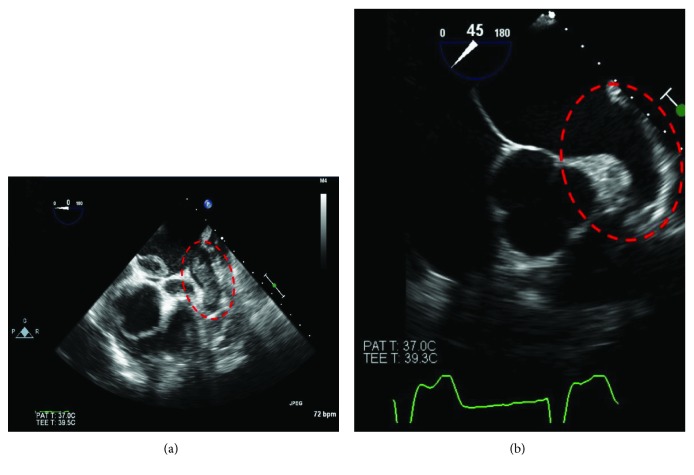
(a) The TEE transesophageal short axis view shows a large multilobulated mobile thrombus in the left atrial appendage. (b) TEE done 3 months later shows resolution of the thrombus.

Transthoracic echocardiogram (TTE) showed left ventricular ejection fraction of 20% and severe global hypokinesis. Coronary angiogram revealed minimal coronary artery disease. The patient was diagnosed with nonischemic cardiomyopathy and was treated with lisinopril, metoprolol, spironolactone, diuretics, and enoxaparin. Despite medical management, he remained in atrial fibrillation for which he was scheduled for rhythm restoration with transesophageal echocardiogram- (TEE-) guided DC cardioversion (DCCV). TEE revealed a large multilobulated mobile thrombus in the left atrial appendage, and sessile irregular echogenic material attached to the wall of the left atrium was visualized (Figure 3(a)). Accordingly, cardioversion was aborted. The patient refused anticoagulation with Coumadin therapy and instead opted for rivaroxaban, aware of risks of possible anticoagulation failure or adverse events, as he would not be on standard of therapy. The patient was discharged with guideline-directed management for coronary artery disease and heart failure as well as rivaroxaban 20 mg daily. On subsequent outpatient follow-up three months later, repeat TEE showed no visible thrombus (Figure 3(b)). No evidence of clinical thromboembolic events was noted between initial and follow-up encounters.

## 3. Discussion

Standard of therapy for stroke prophylaxis in setting of LA/LAA/intracardiac thrombus in patients with NVAF is vitamin K antagonist (VKA) oral anticoagulation [6–10]; however, there is limited data regarding the use and clinical outcomes of direct thrombin inhibitors (DOACs) for diagnosed thrombus in LA/LAA. The ROCKET-AF trial evaluated the treatment of nonvalvular atrial fibrillation (NVAF) with the direct oral factor Xa inhibitor (rivaroxaban) compared with warfarin. Rivaroxaban was noninferior to warfarin in preventing stroke or systemic embolism in NVAF patients [11]. Among patients excluded were those with mitral valve stenosis, prosthetic valves, and left ventricular thrombus; however, there is no specific mention of inclusion or exclusion of LA/LAA thrombus. Investigators commented that patients in the warfarin group were in the therapeutic INR range, a mean of 55% of the time [11].

According to the 2018 European Heart Rhythm Association Practical Guide on the use of nonvitamin K antagonist oral anticoagulants in patients with atrial fibrillation [12], in patients with atrial fibrilation ≥ 48-hour duration (or unknown) provided TEE negative for thrombi, the initiation of DOACs can be considered with at least a single DOAC dose ≥ 4 hours before electrical or pharmacological cardioversion. Otherwise, an alternative strategy would be initiating DOAC therapy for at least 3 weeks prior to cardioversion. After cardioversion, continuous oral anticoagulation is mandatory for at least another 4 weeks, irrespective of the CHA2DS2-VASc score [7]. One prospective open-label multicenter study by Lip et al. (X-TRA study) explored the use of vitamin K antagonist oral anticoagulation for the treatment of nonvalvular atrial fibrillation/flutter with documented LA/LAA thrombi on TEE [13]. This was based on the 2015 CLOT AF registry which with a total of 156 sample sizes in ITT population at 23 institutions among 7 European country [14] findings from the X-TRA showed that resolved or reduced thrombus after rivaroxaban treatment was evident and consistent with LA/LAA thrombus resolution with VKA therapy, suggesting that rivaroxaban maybe a potential treatment option for LA/LAA thrombi in patients with NVAF or atrial flutter [13].

Warfarin inhibits the hepatic synthesis of the vitamin K-dependent coagulation factors by preventing extension of existing thrombi and de novo thrombosis [8]. Limitations for use of warfarin among cardiac patients who require anticoagulation therapy include a narrow therapeutic range, food and drug interactions, and unpredictable pharmacokinetics requiring regular and frequent International Normalized Ratio (INR) checks [15]. Failure of adequate anticoagulation can lead to progression of thromboembolism, hemorrhage, repeated hospital admissions, necessitation of more invasive procedures such as placement of a left atrial appendage closure device, increased cost burden, and increased risks of morbidity and mortality [9, 16, 17].

Rivaroxaban is approved for the reduction in risk of stroke for NVAF, primary and secondary venous thromboembolism (VTE) prophylaxes, and treatment of VTE; however, limited data and literature have been reported to establish the use or efficacy of DOACs on established left-sided intracardiac thrombus and its outcomes. This case demonstrates successful resolution of LA/LAA thrombi on DOAC therapy, specifically rivaroxaban. If enough clinical data supports DOAC relative efficacy in lysis of LA/LAA/intracardiac thrombus, the benefits of DOAC compared to the standard of therapy could increase patient compliance, reduce length of stay, and improve treatment efficacy.

## 4. Conclusion

At present time, there is limited data on DOACs or factor Xa inhibitors in cases of diagnosed left atrial/left atrial appendage/intracardiac thrombi. Ideally, a well-designed randomized clinical trial to provide meaningful data and guidance as to the use of DOACs for such cases.

## References

[1] Chugh S. S., Havmoeller R., Narayanan K. (2014). Worldwide epidemiology of atrial fibrillation: a Global Burden of Disease 2010 Study. *Circulation*.

[2] Brickner M. E., Friedman D. B., Cigarroa C. G., Grayburn P. A. (1994). Relation of thrombus in the left atrial appendage by transesophageal echocardiography to clinical risk factors for thrombus formation. *The American Journal of Cardiology*.

[3] Kamp O. (1999). Importance of left atrial appendage flow as a predictor of thromboembolic events in patients with atrial fibrillation. *European Heart Journal*.

[4] Ruff C. T., Giugliano R. P., Braunwald E. (2014). Comparison of the efficacy and safety of new oral anticoagulants with warfarin in patients with atrial fibrillation: a metaanalysis of randomised trials. *The Lancet*.

[5] Salazar C. A., Aguila D. D., Cordova E. G. (2014). Direct thrombin inhibitors versus vitamin K antagonists for preventing cerebral or systemic embolism in patients with non-valvular atrial fibrillation. *Cochrane Database of Systematic Reviews*.

[6] Developed with the special contribution of the European Heart Rhythm Association (EHRA), Endorsed by the European Association for Cardio-Thoracic Surgery (EACTS), Authors/Task Force Members (2010). Guidelines for the management of atrial fibrillation: The Task Force for the Management of Atrial Fibrillation of the European Society of Cardiology (ESC). *European Heart Journal*.

[7] Kirchhof P., Benussi S., Kotecha D. (2016). 2016 ESC Guidelines for the management of atrial fibrillation developed in collaboration with EACTS. *European Heart Journal*.

[8] Corrado G., Tadeo G., Beretta S. (1999). Atrial thrombi resolution after prolonged anticoagulation in patients with atrial fibrillation. *Chest*.

[9] Egolum U. O., Stover D. G., Lenihan D. (2013). Intracardiac thrombus: diagnosis, complications and management. *The American Journal of the Medical Sciences*.

[10] Delewi R., Zijlstra F., Piek J. J. (2012). Left ventricular thrombus formation after acute myocardial infarction. *Heart*.

[11] Patel M. R., Mahaffey K. W., Garg J. (2011). Rivaroxaban versus warfarin in nonvalvular atrial fibrillation. *New England Journal of Medicine*.

[12] Steffel J., Verhamme P., Potpara T. S. (2018). The 2018 European Heart Rhythm Association Practical Guide on the use of non-vitamin K antagonist oral anticoagulants in patients with atrial fibrillation. *European Heart Journal*.

[13] Lip G. Y., Hammerstingl C., Marin F. (2016). Left atrial thrombus resolution in atrial fibrillation or flutter: results of a prospective study with rivaroxaban (X-TRA) and a retrospective observational registry providing baseline data (CLOT-AF). *American Heart Journal*.

[14] Lip G. Y. H., Hammerstingl C., Marin F. (2015). Rationale and design of a study exploring the efficacy of once-daily oral rivaroxaban (X-TRA) on the outcome of left atrial/left atrial appendage thrombus in nonvalvular atrial fibrillation or atrial flutter and a retrospective observational registry providing baseline data (CLOT-AF). *American Heart Journal*.

[15] Dobashi S., Fujino T., Ikeda T. (2014). Use of apixaban for an elderly patient with left atrial thrombus. *BMJ Case Reports*.

[16] Agarwal S., Hachamovitch R., Menon V. (2012). Current trial-associated outcomes with warfarin in prevention of stroke in patients with nonvalvular atrial fibrillation: a meta-analysis. *Archives of Internal Medicine*.

[17] Mearns E. S., White C. M., Kohn C. G. (2014). Quality of vitamin K antagonist control and outcomes in atrial fibrillation patients: a meta-analysis and meta-regression. *Thrombosis Journal*.

